# The Effect of Verbal Encouragement on Performance and Muscle Fatigue in Swimming

**DOI:** 10.3390/medicina58121709

**Published:** 2022-11-23

**Authors:** Luca Puce, Carlo Trompetto, Antonio Currà, Lucio Marinelli, Laura Mori, Marco Panascì, Filippo Cotellessa, Carlo Biz, Nicola Luigi Bragazzi, Pietro Ruggieri

**Affiliations:** 1 Department of Neuroscience, Rehabilitation, Ophthalmology, Genetics, Maternal and Child Health (DINOGMI), University of Genoa, 16132 Genoa, Italy; 2 Istituto di Ricovero e Cura a Carattere Scientifico (IRCCS) Ospedale Policlinico San Martino, 16132 Genoa, Italy; 3 Academic Neurology Unit, Ospedale A. Fiorini, Terracina, Department of Medical-Surgical Sciences and Biotechnologies, Sapienza University of Rome, Polo Pontino, 04019 Terracina, Italy; 4 Department of Experimental Medicine, Section of Human Physiology, University of Genoa, 16132 Genoa, Italy; 5 Centro Polifunzionale di Scienze Motorie, University of Genoa, 16132 Genoa, Italy; 6 Orthopedics and Orthopedic Oncology, Department of Surgery, Oncology and Gastroenterology (DiSCOG), University of Padova, 35128 Padova, Italy; 7 Laboratory for Industrial and Applied Mathematics (LIAM), Department of Mathematics and Statistics, York University, Toronto, ON M3J 1P3, Canada

**Keywords:** surface electromyography, physiological responses, verbal feedback, motivation, elite athletes, performance optimization

## Abstract

*Background and Objectives:* Verbal encouragement (VE) can be used to enhance performance in several sports, even though no studies have been conducted among swimmers and only a few effects have been reported in elite athletes. Besides influencing motor performance, VE is also known to enhance the physical load, thus potentially increasing the probability of developing fatigue. With this in mind, this study aimed to explore the effects of VE in swimmers in order to fill in the knowledge gap concerning the aquatic environment. *Materials and Methods:* Each athlete swam a maximal 200 m freestyle trial under two different conditions: one trial with VE and the other without VE. The two main outcome measures were: (1) performance velocity (m/s); and (2) muscle fatigue, investigated by means of surface electromyography. Sixty swimmers were recruited, aged 18.63 ± 3.46 years (median 18 years), 28 men (47%), and 32 women (53%), with 7.03 ± 3.9 years of experience. *Results:* With VE, performance significantly improved in the swim trial (*p* < 0.001, effect size (ES) −0.95, large). When breaking the results down into the first half (first (0–100 m) vs. the second half (100–200 m)), the ES was large in the first part (−1.11), indicating an improvement in performance. This worsened, however, in the second part of the trial (ES 0.63). In the multivariate analysis, years of experience were found to be a significant predictor of the change in overall performance (*p* = 0.011). There was a significant increase in muscle fatigue induced by VE, overall, and during the second half, but not during the first half of the trial. *Conclusions:* The present study indicates that VE during a middle-distance event (200 m) increases performance most in swimmers with little experience. However, it has a negative impact on fatigue.

## 1. Introduction

In most athletic disciplines, coaches adopt verbal encouragement (VE) to motivate athletes and improve their motor performance [1,2,3,4,5,6,7,8,9,10,11,12,13]. Generally, in relation to the duration of the exercise, VE has been proposed with a frequency of 5 [4] or 10 s [11] and, in some cases, every 20, 60, or 180 s [5]. In other studies, the exact VE modality was not predefined/specified [6,7]. Words and expressions utilized were almost always standardized [13] and delivered by the coach, and the volume was slightly higher than what is commonly used during a conversation [9,12]. Sometimes the voice was recorded and heard by the participants via headphones [11].

VE proved beneficial in several land sports and under various experimental conditions [1]; however, it has never been studied in an aquatic environment, probably due to the technical difficulties of transmitting clearly audible and understandable sounds underwater. In the extant scholarly literature, subjects encouraged by motivational feedback during a fatigue task on a treadmill improved their endurance and sprint performance [2,3]. In addition, greater strength was found in dynamic activities carried out in football, tennis, cycling, and running [4,5,6,7], as well as during the isometric and concentric muscle contractions performed in other disciplines [8,9,10,11].

In contrast, a few studies failed to confirm VE’s beneficial effect. For example, the maximum isometric hand grip strength in senior male judokas did not increase [12], the upper body performance in elite rugby players remained essentially unchanged (with only small variations) [13], and high-intensity functional strength and endurance tasks in “expert functional athletes” did not differ with or without VE [14]. Interestingly, the participants in these studies were elite athletes (with high levels of experience and performance).

Besides influencing motor performance, VE is also known to increase the physical load. For example, in soccer players and school students engaged in different modalities of small-sided games (3 vs. 3, 4 vs. 4, 5 vs. 5, and 6 vs. 6), VE increased the heart rate (HR), the blood lactate concentration (LA), and the rating of perceived exertion (RPE) [15,16,17]. Likewise, during training sessions, VE increased the HR and RPE in young tennis players [4]. Finally, VE increased both the maximal oxygen uptake (VO_2max_) and HR during a 20 m multistage shuttle run test [18].

By increasing the physical load, VE increases the probability of developing fatigue [5], which can be defined as an exercise-induced reduction in the muscle’s capability to generate force [19]. Therefore, fatigue could alter the physical and cognitive functions of the athlete and become a limiting factor in the effectiveness of the VE [20]; however, as far as we know, no attention has been paid to this issue so far.

Real-time monitoring of the fatigue affecting a particular muscle while performing an activity is possible via surface electromyography (sEMG) [21]. Indeed, the biochemical changes in the muscles (accumulation of catabolites, such as inorganic phosphate and phosphocreatine) during strenuous contractions are also reflected in the properties of the myoelectric signal generated. A greater concentration of the power spectrum of the sEMG signal around the lower frequencies corresponds to a decreased motor unit discharge rate and a change in the shapes of the motor unit action potentials that compose the sEMG signal [22]. These complex electrophysiological phenomena are referred to as localized muscle fatigue [23]. The median frequency of the power density spectrum (MF [Hz]) (defined as the frequency value that divides the power spectrum of the sEMG signal into two sections of equal energy content) provides a reliable estimate of the frequency shift [24]. Furthermore, compelling evidence shows that its progressive decrease during the entire exercise is evident and indicative of myoelectric fatigue [25].

The aim of this research was to study VE in swimmers by evaluating both chronometric performance and muscle fatigue (using sEMG) to understand and identify the effects of this method in order to fill in the knowledge gap concerning the aquatic environment. Based on previous reports [1,2,3,4,5,6,7,8,9,10,11,12,13,14,15,16,17,18,19,20], it was hypothesized that VE would improve swimming performance at the cost of increased fatigue.

## 2. Methods

### 2.1. Recruitment of Participants

The swimmers were recruited at the Albaro swimming pool center in Genoa, Italy. The inclusion criteria were: (1) at least 1 year of experience in training for regional and international competitions; (2) a score of ≥ 200 points on their personal best in the 200 m freestyle, in accordance with the international point score (IPS) [26]. The IPS system allows the comparison of the performance levels between swimmers of different sexes and ages by assigning point values to their swimming performance: more points for world-class performances (typically 1000) and fewer points for slower performances. Exclusion criteria were the presence of muscle pain or soreness capable of compromising the athlete’s ability to express maximum strength.

Written informed consent was obtained from all subjects prior to their participation in the study in accordance with Resolution 466/2012 of the National Commission for Research Ethics (CONEP) of the National Health Council and the ethical principles expressed in the Helsinki Declaration 2014 by the World Medical Association (WMA) for experiments involving humans. This randomized, counterbalanced cross-over study was registered (Protocol Record: VES-DINOGMI-21, Identifier: NCT05573971) and approved by the local ethics committee of the University of Genoa, Genoa, Italy (No. 2020/21).

### 2.2. Characteristics of the Sample

Of the 75 screened swimmers, 60 met the inclusion criteria and were enrolled in the study. They were 28 men (47%), and 32 women (53%), aged 18.63 ± 3.46 years (median 18 years), with 7.03 ± 3.9 years of experience in training for regional and international competitions. The mean height was 175.17 ± 7.90 cm, the mean body mass was 65.23 ± 10.11 kg, and the body fat percentage was 11.45 ± 1.84%. Their best performance in the 200 m freestyle in the long course (50 m) swimming pool was on average 134.26 ± 19.22 s, corresponding to 716 ± 164 points (median 746) of the 50 m pool world record, in accordance with the IPS (Table 1).

### 2.3. Anthropometric Measurements

An impedance balance (Omron BF 511, Hoofddorp, The Netherlands) with a capacity of 0 to 150 kg and an accuracy of 0.05 kg was used to determine body mass and body fat measurements. The height was measured with a free-standing stadiometer without footwear, with the participants’ heels and backs touching the wall.

### 2.4. Experimental Design

The study was designed as a randomized, counterbalanced crossover trial. Each athlete swam a maximal 200 m freestyle trial under two different conditions: one trial with VE and the other without VE. Both trials were performed at the same time of day with a one-week interval between them. The sequence order of the swimming trial conditions (with VE plus without VE/without VE plus with VE) was randomly generated by a computer (complete randomized across-subjects counterbalanced/between-subjects counterbalanced design). The outcome measures of this study were: swimming velocity (m/s) to evaluate performance and MF [Hz] as an indicator of muscle fatigue.

### 2.5. Swim Trial

After the personalized warm-up (≈15 min low-intensity aerobic swimming, ≈10 min drill exercises, and ≈10 short sprints), the swimmers were instructed to perform a 200 m front crawl at maximum velocity in an indoor 50 m pool with a water temperature of ≈28 °C. Due to the sEMG equipment and markers attached to the body, participants started swimming in the water with a push-off from the wall.

VE was provided by the same coach through Swim Coach Communicator (FINIS Swimming, Livermore, CA, USA). The communicator works with a Bluetooth connection that starts from the coach’s smartphone and arrives at the swimmer by attaching two devices to the elastic of the athlete’s goggles. The two devices were positioned at the temples and transferred sound via vibrations from the temporal bone to the cochlea.

“Swim as fast as you can! Go! Go! Go!” and “Stronger! Stronger! Stronger! Do not give up!” were used as VE at the 15^th^ and 45^th^ meters, respectively, of each lap (50 m) of the 200 m trial. The temporal frequency of VE throughout the test was chosen based on a previous study [5].

### 2.6. Kinematic Assessment

The swim test was videotaped in the sagittal plane using two cameras (GoPro Hero 8 model, GoPro, San Mateo, CA, USA), one above the water surface and the other below.

The cameras were attached to a pushcart which was moved at the same speed as the participants.

The video recording was manually synchronized with the sEMG trace through a tapper on the sensor recognizable from both the video and the sEMG trace. Information on the position of the body and limbs of the swimmer was obtained by applying adhesive markers on the joints of their lower and upper limbs. The kinematic evaluation made it possible to calculate the total time of 200 m, the split times for every 100 m (first (0−100 m) and second half (100−200 m)), and to distinguish the underwater phase of each stroke for the sEMG assessment.

The arm extended forward underwater at the end of the previous arm recovery coincided with the start of the underwater phase. The exit of the arm from the water at hip height, with the elbow leading and the forearm and hand following, coincided with the end of the underwater phase.

### 2.7. sEMG Assessment

sEMG signals from *Pectoralis Major* (PM), *Triceps Brachii* (TB), *Latissimus Dorsi* (LD), and *Rectus Femoris* (RF) of the dominant side were recorded through bipolar surface electrodes (Ambu Blue Sensor N, Middelfart, Denmark) positioned according to the Surface Electromyography for the Non-Invasive Assessment of Muscle (SENIAM) guidelines [27]. These muscles were selected based on previously published studies assessing their main function in front-crawl propulsion [28].

The sEMG signals were acquired using wireless EMG equipment (Cometa Srl, Milan, Italy) with a band-pass filter of 1st order in the range of 10–500 Hz and digitized at 2000 samples/s.

Raw sEMG signals were processed with a band-pass Butterworth filter of 4th order in the range of 20–500 Hz and full-wave rectified.

A sEMG threshold, fixed at 15% of the peak sEMG value, was used for determining the onset and offset for each interval of activations corresponding to the underwater phase [29].

Once the activation interval was determined individually for every stroke and muscle, the evaluation of the MF value was carried out. Performing this operation gave a plot of MF value vs. time. To estimate the time evolution of MF, a linear fitting of the data set was performed, and the slope was extracted. The slope was finally normalized to the value of the regression line at the initial time of the first analyzed activation interval for each sEMG trace. Three slopes for each swimming test were expressed as percentage values corresponding to the first part (0–100 m), the second part (100–200 m), and the whole test (0–200 m), respectively. MF for every 50 m was not evaluated to minimize unpredictable variations in the time course of the quasi-cyclostationary myoelectric signal [30].

## 3. Statistical Analysis

A descriptive statistical analysis was conducted. The means with the standard deviations were computed for continuous variables, whereas categorical parameters were expressed as percentages, when appropriate. The Shapiro–Wilk test was conducted to check for the normality of the data distribution. A paired samples Student’s t-test was applied to compare the mean differences with and without VE. Since the statistical significance provides the reader only with the information on whether the intervention (VE) is significant or not, but not in which direction and to which extent [31], in addition to displaying the *p*-value, we also calculated and reported the effect size (ES). For the paired samples Student’s t-test, the ES was computed using Cohen’s d_z_, also known as “the standardized mean difference ES for within-subjects designs” [32]. It is calculated as the ratio of the difference between the two means (observational and interventional, without and with VE) and the standard deviation of the difference scores:

(1) $$Cohen^{\prime }s\;d_{z}=\frac{M_{diff}}{\sqrt{\frac{\sum {\left(X_{diff}-M_{diff}\right)}^{2}}{N-1}}}$$

Note that this is equivalent to Rosenthal’s formula [33,34]:

(2) $$Cohen^{\prime }s\;d_{z}=\frac{t}{\sqrt{N}}$$

This represents the “true” or “real” ES, in that it can effectively capture the variability induced by the VE intervention (i.e., the change in the score standard deviation), which is what is utilized for the computation of the test statistics—that is to say, the t-statistics [35]. The magnitude of Cohen’s d_z_ was interpreted using the following rule of thumb: null/negligible if less than 0.2, small if 0.2–0.5, medium if 0.5–0.8, and large if greater than 0.8 [36]. In the case of a violation in the normality of data distribution, a Wilcoxon signed-rank test (also known as the rank-based version of the paired samples Student’s t-test) was utilized [37]. The ES is conceptually equivalent to the rank biserial correlation [38,39], or to Cliff’s delta, also known as the “dominance statistics” ES [40]. The matched-pairs rank biserial correlation coefficient can be directly computed from the W statistics [41].

Given that:

(3) $$W=\sum_{1}^{N}\left[\mathrm{rank}\left(\left|d_{i}\right|\right)Q\left(d_{i}\right)\right]$$

where $Q\left(d_{i}\right)$ is the sign indicator function of the difference score, defined as [40]:

(4) $$Q\left(d_{i}\right)=\{\begin{array}{c} -1\;if\;d_{i}<0\\ +1\;if\;d_{i}>0 \end{array}$$

$\left(d_{i}\right)$ is the difference score and $\left|d_{i}\right|$ is its absolute value, in such a way that W is the sum of the signed ranks of the difference scores. The matched-pairs rank biserial correlation coefficient can, then, be computed as [41]:

(5) $$r_{c}=1-\frac{4W\;}{N\left(N+1\right)}$$

Note that, in the case of ties, they are removed, and this affects the denominator. Additionally, it should be appreciated that the matched-pairs rank biserial correlation is exactly the rank-based equivalent of Cohen’s d_z_. The magnitude of the rank-based ES was, then, interpreted using the following rule of thumb: 0.147 (small), 0.330 (medium), and 0.474 (large) [42], which is comparable to Mangiafico’s metrics—small (0.1–0.3), medium (0.3–0.5), and large (≥0.5) [36].

Finally, a multivariate regression analysis was conducted to shed light on the determinants of the changes in performance (mean difference) with and without VE. The goodness-of-fit was assessed using R, R-squared (R^2^), adjusted R-squared (aR^2^), Akaike information criterion (AIC), Bayesian information criterion (BIC), root-mean-square error (RMSE), and F-test (overall model fit test). All statistical analyses were conducted using Jamovi v.1.8.2. A cut-off of 0.05 was chosen for statistical significance.

## 4. Results

### 4.1. Impact of VE on Performance

With VE, the performances significantly improved in the swim trial (the mean difference was −0.01 m/s, *p* < 0.001, ES −0.95, large). When breaking down the results into the first half (first (0–100 m) vs. second half (100–200 m)), the ESs were large and medium in the first and second halves (−1.11 and 0.63, respectively), indicating an improvement and worsening in performances, respectively (Table 2).

The fit of the multivariate regression model was satisfactory. In the multivariate analysis, only the number of years of experience was found to be a significant predictor of the change in overall performance (0.002 ± 0.001 (95% CI 0.000–0.003), *p* = 0.011, standardized estimate 0.52 (95% CI 0.12−0.91)) (Table 3).

### 4.2. Impact of VE on Fatigue

In Table 4, we present the fatigue values for each muscle and for each of the two conditions, averaged over the participants. It can be seen, in the swim trial, and during the second half, that there was a significant increase in the fatigue induced by VE (*p* < 0.001). In the first half of the trial, however, no significant differences were found between the two conditions. Specifically, the mean difference in the level of fatigue in the 200 m trial was about 4% in all the types of muscles evaluated. In contrast, for the second half, the mean differences were 3%, 5%, 4%, and 5% in the RF, TB, LD, and PM, respectively.

## 5. Discussion

### 5.1. General Results

The aim of the present study was to explore the effects of VE on muscle performance and fatigue in order to fill in the knowledge gap concerning aquatic environments. Sixty swimmers completed a maximal swim task (200 m freestyle) with and without VE in a randomized counterbalanced order. We observed that VE increased the performances and muscle fatigue to a greater extent in the young swimmers, those with a lower performance level (IPS score), and those with fewer years of experience. Another result we could report is that VE interferes with pacing strategies. In fact, VE increased the performances only in the first half of the swimming task (0−100 m), compromising the second half (100−200 m), due to the greater fatigue experienced in the final stages. However, the overall performance was found to be improved.

### 5.2. Performance and Muscle Fatigue

In accordance with the “10-year rule” (10 years of commitment to high-level training as the minimum requirement to reach the level of an expert) [43], we have found that athletes with approximately 10 years or more of experience had less improvement when verbally encouraged. The achievement of task-specific expertise is, in fact, a long process that requires specific training focused on strength management as a function of the distance to be covered [44].

On the other hand, a lack of experience exposes athletes to be particularly sensitive to external stimuli. This could explain why all athletes with 8 or fewer years of experience had an advantage when verbally encouraged. The IPS also proved to be an important factor, with a trend toward statistical significance. Generally, athletes with a lower IPS benefitted more from VE, even though, when adjusting for all factors, this variable was found not to achieve the significance threshold, because athletes with innate talents can achieve better performance first but remain immature from a technical and tactical point of view. In summary, the level of experience is the most important variable that determines VE sensitivity.

It is worth mentioning that, here, we compare studies of other sports modalities and with other athletes, given the lack of research focused on aquatic environments. In this direction, our results are in line with previous studies.

In adults and experienced athletes practicing functional training, only minimal changes in endurance performance and strength production have been found [14]. These findings have been explained by the fact that the participants had strong intrinsic motivation and, therefore, did not need any external verbal stimulation.

This is in line with what has been found in senior judo athletes, whose maximum isometric hand grip strength showed slightly lower scores during VE [12]. The authors of the study speculated that external stimuli under certain circumstances could interfere with the performance in elite athletes who prefer silence and concentration.

Similarly, Argus et al. [13], who examined the effects of VE on the upper-body performance of rugby players, confirmed this finding by hypothesizing that elite athletes can recruit more motor units than their untrained counterparts, making them less sensitive to external stimuli.

How energy is distributed during exercise can have a substantial impact on performance and is considered crucial for optimal performance in different sports [45]. Specifically, during strenuous and prolonged physical activity, the key to optimally managing performance is achieving a good balance between the anaerobic and aerobic systems, exploiting the increased lactate production only at the “right time” to get the most out of it [46,47]. Conversely, a massive and premature physical load induced by VE led to early fatigue and a deterioration in performance in the second half.

This is particularly relevant in swimming as the relationship between the energy cost and velocity is not linear, given the highly resistive properties of water and the low mechanical efficiency of motions for swimming [47]. However, VE in the frequency mode used in this study interfered with pacing strategies, increasing performances in the first half of the swimming task and decreasing performances in the second half, due to the increase in fatigue. Similar physiological responses were found in the study by Andreacci et al. [5] during a maximal exercise test on a treadmill. The authors found that VE delivered at a short frequency (every 20 s and every 60 s) and already in the initial phase of the test significantly increased the metabolic and cardiovascular variables. Conversely, VE during the third test phase with a higher frequency (180 s) did not reproduce the same effects.

In general, therefore, the fast-start strategy induced by VE proved useful for the swimmers in this study, but it could potentially prove counterproductive during longer events.

### 5.3. Limitations and Strengths

This study is not without any limitations, including the lack of data related to kinematic parameters, such as the stroke length, stroke rate, or stroke index, which are indicators of the effectiveness of a swimmer’s stroke [48]. Furthermore, data concerning physiological responses and parameters related to fatigue, such as HR, LA, VO_2max_, or RPE, were not collected. The small sample size, which may have led to a lack of statistical power, should also be considered a limitation. On the other hand, the study design represents a strength (with the trials with VE and without VE conducted in a randomized controlled order). Moreover, this study is the first to explore the effects of VE on the performance and muscle fatigue of swimmers, probably due to the technical difficulties of perceiving stimuli in the water.

## 6. Conclusions and Practical Applications

The present study indicates that VE during a middle-distance event (200 m freestyle) increases performances most in swimmers with little experience and low performance levels. However, it has a negative impact on fatigue.

VE could be a useful method to improve the quality of training by allowing better physiological adaptations. Nonetheless, further studies are needed to better support this preliminary recommendation in a data-driven and evidence-based fashion.

## Figures and Tables

**Table 1:** Main demographic and anthropometric features of the sample. Abbreviation: IPS, International Point Score.

Parameter	Value
Sex (number, percentage)	
Male	28 (47%)
Female	32 (53%)
Age (mean, standard deviation (median))	18.63 ± 3.46 (18) years
Height (mean, standard deviation)	175.17 ± 7.90 cm
Body mass (mean, standard deviation)	65.23 ± 10.11 kg
Body fat (mean, standard deviation)	11.45 ± 1.84%
Personal best (mean, standard deviation)	134.26 ± 19.22 s
IPS (mean, standard deviation (median))	715.87 ± 163.71 (746) points
Experience (mean, standard deviation (median))	7.03 ± 3.90 (6) years

**Table 2:** Performance with and without verbal encouragement (VE), and changes in performance broken down according to the distance. Abbreviations: ↑ improved; ↓, worsened; CI, confidence interval; SE, standard error; ES, effect size.

Distance	Without VE	With VE	Statistics (*p*-Value)	Mean Difference ± SE [95% CI]	ES [95% CI]	Interpretation [Direction (Magnitude)]
0–200 m	1.47 ± 0.17 m/s	1.48 ± 0.17 m/s	−7.33 (<0.001)	−0.011 ± 0.002 (−0.014 to −0.008)	−0.95 (−1.25 to −0.64)	↑ (large ES)
0–100 m	1.50 ± 0.17 m/s	1.53 ± 0.15 m/s	−8.60 (<0.001)	−0.033 ± 0.004 (−0.041 to −0.025)	−1.11 (−1.43 to −0.78)	↑ (large ES)
100–200 m	1.44 ± 0.17 m/s	1.43 ± 0.18 m/s	4.89 (<0.001)	0.008 ± 0.002 (0.004 to 0.011)	0.63 (0.35 to 0.91)	↓ (medium ES)

**Table 3:** The multivariate regression analysis sheds light on the determinants of the change in performance due to verbal encouragement. Abbreviation: AIC, Akaike information criterion; aR^2^, adjusted R-squared; BIC, Bayesian information criterion; CI, confidence interval; F-test, overall model fit test; IPS, International Point Score; R^2^, R-squared; RMSE, root-mean-square error; SE, standard error.

Predictor	Estimate	SE [95% CI]	Statistics (*p*-Value)	Standardized Estimate [95% CI]
Intercept	−0.036	0.011 (−0.057 to −0.014)	−3.26 (0.002)	-
Sex (female vs. male)	−0.003	0.002 (−0.008 to 0.001)	−1.68 (0.099)	−0.30 (−0.66 to 0.06)
Age	0.000	0.001 (−0.001 to 0.001)	0.46 (0.647)	0.07 (−0.24 to 0.38)
IPS	0.000	0.000 (0.000 to 0.000)	1.62 (0.112)	0.22 (−0.05 to 0.49)
Years of experience	0.002	0.001 (0.000 to 0.003)	2.64 (0.011)	0.52 (0.12 to 0.91)
R = 0.74, R^2^ = 0.55, aR^2^ = 0.52, AIC = −403, BIC = −391, RMSE = 0.008, F_(4,55)_ = 16.9, *p* < 0.001				

**Table 4:** Abbreviation: ES, effect size; LD, *Latissimus Dorsi*; PM, *Pectoralis Major* RF, *Rectus Femoris*; TB, *Triceps Brachii*; VE, verbal encouragement.

Muscle	Distance (m)	Without VE (%)	With VE (%)	Mean Difference (%)	Statistics (*p*-Value)	ES (Magnitude)
RF	0–200	−6.40 ± 4.00	−10.28 ± 3.14%	4.03 ± 0.51	1656 (<0.001)	0.87 (large)
	0–100	−3.78 ± 2.35	−3.62 ± 2.12	0.04 ± 0.13	811 (0.446)	0.11 (null/negligible)
	100–200	−8.45 ± 4.47	−11.42 ± 3.28	3.07 ± 0.39	1622 (<0.001)	0.83 (large)
TB	0–200	−10.77 ± 5.48	−15.35 ± 4.68	4.23 ± 0.63	1751 (<0.001)	0.91 (large)
	0–100	−10.03 ± 6.19	−10.55 ± 4.94	0.07 ± 0.42	789 (0.609)	0.08 (null/negligible)
	100–200	−14.14 ± 6.32	−19.88 ± 6.00	5.08 ± 0.85	1720 (<0.001)	0.88 (large)
LD	0–200	−10.58 ± 3.81	−15.00 ± 5.88	4.46 ± 0.56	1639 (<0.001)	0.85 (large)
	0–100	−9.37 ± 4.54	−9.81 ± 3.89	0.20 ± 0.27	1147 (0.048)	0.30 (small)
	100–200	−14.88 ± 4.31	−19.34 ± 6.30	4.39 ± 0.53	1673 (<0.001)	0.83 (large)
PM	0–200	−9.62 ± −4.75	−13.31 ± 2.89	3.63 ± 0.48	1713 (<0.001)	0.87 (large)
	0–100	−5.62 ± 1.76	−5.37 ± 1.66	0.22 ± 0.15	663 (0.064)	0.28 (small)
	100–200	−14.11 ± 3.55	−18.73 ± 2.27	4.71 ± 0.45	1765 (<0.001)	0.93 (large)

## Data Availability

Not applicable.
